# Healthy Architecture! Can environments evoke emotional responses?

**DOI:** 10.5539/gjhs.v4n4p83

**Published:** 2012-06-05

**Authors:** Kirsten Kaya Roessler

**Affiliations:** 1 Department of Psychology, University of Southern Denmark, Faculty of Health Sciences, Odense, Denmark

**Keywords:** architecture, environmental psychology, public health, single cases

## Abstract

We find environmental psychology at the intersection between architecture and psychology. This article discusses the ways in which individuals are affected by architecture, departing from an early source on the psychology of architecture and taking three architectural examples as illustrations: a public place in Berlin, a health environment in Sweden, and a fitness centre in Denmark. Each of these architectural examples creates what might be called its own psychological emotions, and these are analysed and discussed using a psychodynamic and existential attempt to understand the interrelationship between individuals and spatial reality. A health oriented existential approach is used as a methodological basis to conceptualise the psychological effects of various forms of architecture.

## 1. Introduction

### 1.1 Environmental Psychology

Since the early 1960’s we find *environmental psychology* as overlap between architecture and psychology (Altman & Wohlwil, 1977; Canter, 1974). The discipline started, when a research team investigated the architecture of psychiatric hospitals in order to create a *healing hospital* and to underpin the therapeutic work with the patients (Ittelson, 1960; Lazarus, 1977). This soon extended to asking questions about the general relationship between space, and people. The relationship between space and social behaviour (Altman, 1975) covered people’s need for intimacy and privacy (Hall, 1966). Also the suitability of the city for human habitation without alienation was a theme of this early research phase (Mitscherlich, 1971 & 1972; Kükelhaus, 1983).

Meanwhile, the research in environmental psychology has developed into a range of different themes and issues. Knowledge in environmental psychology is becoming more and more important for public health (Annerstedt & Währborg, 2011), designing of hospitals (Janssens, 2001), and urban areas (Hannes et al., 2009).

The present study aims to take a step back and to assess the influence of three environments on the observer’s emotions and perceptions. Aim of the paper is to investigate how (and if) one from a psychological point of view can describe whether these single case environments have an impact on health related existential aspects such as isolation or meaning.

### 1.2 Impression and Expression: A Historical Framework

Almost a century before the discipline of *environmental psychology* was established, a young Swiss critic and later professor of art, Heinrich Wölfflin (1886) wrote a dissertation entitled *Prolegomena to a psychology of architecture* [Prolegomena zu einer Psychologie der Architektur]. He attempted to show that architectural forms are *expressions* for certain spirits or moods. Buildings could give the impression of being serious or sombre or friendly, but it was difficult to explain why. Wölfflin aimed to explain how the experienced *impression* of a building is mirrored by the *expression* of the object. He was claiming that psychologically architecture had its basis in form through the empathetic response of human form.

“Our corporeal organisation is the form through which we experience everything physical. I will now show, that the basic elements of architecture: material and form, weight and power, are determined by the experiences we have had”^1^

Wölfflin’s text is considered to be one of the founding texts of the emerging discipline of architectural and art psychology. He showed that the main idea of architecture was gravity and inflexibility supported by the proportions of length and breadth along horizontal or vertical plane. Public buildings (for example theatres or libraries) can be characterized by breadth; the buildings have extension while a tower has height. The relationship between extension and height determines whether a building seems to be awkward, light, flexible or static. An explanation from out concepts such as form, material and proportion is focused on the object and its expression.

Wölfflin’s idea was that a certain expression provokes a certain impression. He found elements of the human body – such as weight, form or power – represented in architecture. Taking root in this observation, he described the impression of a building being affected by its “bodily” expression. While Wölfflin mostly argued from the *expression* of the building or environment, this study tries something different. It looks at the *impression*, as the way subjects experience an architectural object. In order to analyse a given space or to obtain an overall perspective of the factors relevant to people’s experience of a given space, Wölfflins approach shall be extended towards the impression.

## 2. Methods and Research Questions

The study employed qualitative methods. Qualitative research was chosen as appropriate method of obtaining first hand experience. Three cases are in this pilot study analyzed and compared from out the impression of the observer. The research methodology is understood as *participatory research*, arguing “in favor of the possibility, the significance, and the usefulness of involving research partners in the knowledge-production” (Bergold & Thomas, 2012). The cases involve an in-depth examination of a single instance or event. They provide a subjective way of looking at events, collecting data, analyzing information, and reporting the results. The cases are related to three different environments: a public place in Germany, a health environment in Sweden and a fitness studio in Denmark. The three cases are selected based on their diversity (Flybjerg, 2006) and do not allow a generalization. They try to include a variation of indoor and outdoor environments, and city and countryside environments. The three cases are used to develop a general theoretical approach.

Aim of the paper is to discuss subjective emotions in interrelation with architecture. The research question to be answered is, whether certain environments can provoke or reinforce emotional impressions and support health behaviour. Three quite different and fortuitous cases – a garden, a place and a fitness centre – are chosen as pilot environments. It’s a work hypothesis that environments *per se* are capable of evoking a certain emotional response. The study is a pilot study or almost a reflective essay to develop questions to further research.

## 3. Analysis and Results

### 3.1 Potsdamer Platz, Berlin, Germany

Berlin’s *Potsdamer Platz* is a monument to the modern art of construction and a place in which large numbers of people move about at any given time. Wölfflin would probably describe the appearance of the place as enlightening, where buildings are *standing* (height > breadth) not *lying* (breadth > height) and the atmosphere seems to be light, not symmetric, but flexible.

*“The square seems gigantic in its structure. It seems to be the central of the world, a main traffic point in new city Berlin. This is a place, where economic ambitions show their manifestation as skyscrapers. The Potsdamer Platz seems to be a major redevelopment project. It is transparent and unambiguous in its structure, but I can’t find opportunities to withdraw and experience a sense of safety. Crossing the Potsdamer Platz, I’m constantly visible and have nowhere to hide. I feel alone and alienated even if I know, that I am constantly surrounded by other humans*” (Author, Berlin).

Two famous German psychoanalysts were already during the 1960’s discussing the question of alienation being an outcome of modern architecture (Lorenzer, 1960; Mitscherlich, 1972). Alfred Lorenzer described the emotional isolation of the individual as a symptom and a pathological result of functional architecture that prefers aesthetics instead of meaning. He was followed up by Alexander Mitscherlich, who talked about lack of hospitality as a symptom of modern cities.

It is tempting to discuss the *interpersonal* isolation as relationship between the expansiveness of this space and the lack of continuity generated by the square. The name “Potsdam” awakens historical associations not only to the Prussian period but also to the rupture of war and the Potsdam Conference of 1945, when Germany was divided into three zones. But this history exists nowhere in the square, the towers of capitalism, like the Sony tower, are empty of history. They remain isolated and alone, especially if we compare them to the old tradition of Berlin’s historical buildings. A historical building, for example the “*Oper unter den Linden*” exudes the feeling of being in close contact with history, while the Potsdamer Platz signals separation and offers no refuge. Such separation can evoke anxiety such as agoraphobia. Freud noted that “in the case of agoraphobia etc. we often find the recollection of an anxiety attack; and what the patient fears is the occurrence of such an attack under the special conditions in which he believes he cannot escape it” (1962, p. 81).

### 3.2 Healing Gardens, Alnarp, Sweden

A contrasting picture appears at Alnarp’s Healing Gardens. In the small Swedish town Alnarp near Malmö, researchers (Stigsdottir & Grahn, 2002, 2010) discovered that experiences of nature affect people differently, largely depending on their life situation. Natural environments are particularly helpful in restoring attention, as they provide gently stimulation to the senses and offer a range of sights, smells and sounds (Kaplan, 1989). Kaplan argues that the “soft fascination” of natural settings can enhance the recovery from stress disorders, and that health and well-being can be promoted by green spaces.

The main expression of Alnarp Garden is coherence. The garden is on an enclosed ground and consists mainly of living organic material such as plants and wood. The garden offers a great variety of sensory stimulations and experiences.

“*I want to discover the garden on my own. Go around, sneeze the plants, find secret corners. The garden invites me to move, to walk around, look and touch. Being in the garden, I wish to participate and to be active. I want to plant or nurse flowers or to sit in a quiet corner and to enjoy the open landscape. Sitting in a quite corner, I feel safe and relaxed. I can decide myself whether I look for the old rabbit, become involved in planting or just relax under a tree*” (Author, Alnarp).

Alnarp’s Healing Garden is a meeting place including research and education at the Faculty of Landscape Planning, Horticulture and Agriculture of Malmö University. Here, different types of research and development attempts are carried out. An interdisciplinary team works with stress patients using the possibilities of communication offered by garden spaces with different characters. The garden communicates to the individual on different levels connected to different spaces, colours or plants. The garden is a space for movement, a manifestation of life, and applied art. The main theme of the garden is *participation*, and to change from *active* participation to *emotional* participation or *inwards* involvement (Stigsdottir & Grahn, 2002). The aim of the design was to create garden spaces, which were not too abstract, unfamiliar or challenging. While the Potsdamer Platz mostly appealed to the aspect of isolation and ahistorical separation, Alnarp’s Gardens offer a more complex mix of aspects. Following the plants through different periods of the year, nursing and planting of flowers will support a quality of responsibility and willing. Responsibility includes both the word answer (response) and ability. In Alnarp it is for example possible to take responsibility for the two old rabbits, which one may take on one’s arm, feed or carry around.

### 3.3 A Fitness Studio

The last example is a fitness studio in Denmark. The expression of the architecture is functional; the building has no ornaments or decorations on the facade. Inside, the space is dominated by the fitness machines, which fill most of the area of the room. The ruling colors are silver metallic and black, and the carpet is gray.

*“Coming from a dark parking space outside, where I feel frightened, I enter facilities that are full of light. From the neon enlightened indoor room with the big floor to loft glass windows, I feel being watched by a foreign and unknown audience. I feel bothered by shame when doing physical activity and I like to be private. A lot of physical activity is on the floor and needs to be in a private atmosphere”* (Female, 58 years, Odense).

This quote is part of a Danish study of female and male expectations with regard to architecture related to movement (Munch, Mogensen, & Roessler, 2007; Roessler, 2007). The investigation was based on the hypothesis that there exist gender differences concerning expectations, barriers and wishes regarding indoors and outdoors architectural facilities. The research results, which will not be described here in detail, showed that architecture, which reinforced loose of control and supported embarrassment especially influenced female exercising behaviour. Men and women showed significant differences in the psychological factors associated with sports participation. For women’s exercising behaviour were the fear of violation and the feeling of insecurity on a dark running track, important barriers to their outdoor physical activities. When exercising indoors, awkwardness was frequently named as a barrier. For example, when lying on their backs doing Pilates exercises, women feel more exposed to an observer’s gaze than when weights training. Thus, women feel a need for privacy and safety, because some positions and movements make them feel more vulnerable and visible in their surroundings. Here, for example, the light from an un-curtained window is viewed as frightening rather than ‘enlightening’.

Women feel in general more exposed to the observer’s gaze in a different way. There is a need for privacy and safety, because some movements make people more vulnerable and visible to their surroundings.

“*In our gym, the spinning bikes are closely positioned beside each other. This can be felt like an intrusion of one’s personal space, but it can conversely have a motivating and performance-enhancing effect – particularly when people are engaged in an activity such as spinning. A spinning room is for me a ‘safe’ place, where one is able to predict what will happen”* (Female, 43, Copenhagen).

A sports environment can offer a challenging opportunity both indoors and in a densely populated area. In this context, ‘challenge’ means that movements are not immediately through architecture squeezed into a particular framework but allow freedom to develop. A space is considered *challenging* if one wants to test it right away – to invade it, feel the surface, climb up, slide down or run across it. Whether or not a space is challenging depends on many aspects, including people’s age, gender, knowledge and interests.

## 4. Discussion

The research question for this study was – outgoing from the historical text of Wölfflin – whether certain environments can provoke or reinforce certain emotions. The three described environments separated or linked activities, organised social contacts, privacy, overview and aesthetics in very different ways.

Asking for emotions is related to existential aspects such as fear, need or motivation. Following an existential psychological approach, the impression of a given environment is interpreted as an attempt to tackle, reduce or overcome the anxiety that emanates from an awareness of the finite nature of existence (Yalom, 1980). Anxiety is seen as central driving force in life. According to Yalom’s theory, the individual evolves through four existential themes: finality, responsibility, isolation and meaninglessness. Human beings are unique, but have also to be capable of opening up to the world. We are referred to adulthood confronted with a paradox: we need to maintain and realise our self, but we also have to be able to give of and forget our self. We have to overcome the fear of infinity. The fear of isolation becomes a fixed part of life with an impact on health – people fear both being alone and being with others. Later in life the fear of infinity and death may conflict with the desire for responsibility and autonomy. Overall, there is a striving after meaning, a desire to sink roots and plan for the future (Yalom, 1989). In other words, the individual faces another paradox: s/he has to strive not only after continuity, but also change. The individual exists in a state of tension between its fears of *death, freedom, isolation* and *meaningfulness*. Throughout our lives, different types of anxiety dominate – in some periods we are afraid of being alone, while in others we seek for meaning.

Let us now try to interpret the three case environments concerning their impact on health following Yalom’s approach. The *Potsdamer Platz* can be analysed as a place that generates *isolation*. Yalom differs isolation into intrapersonal, interpersonal and existential (Yalom, 1980). For the observator, this place seems to generate existential isolation, defining a chasm between the individual and other human beings. Vast glass frontages, high towers and large amounts of neon light make the individual feel small and isolated. There is a lack of privacy (Hall, 1966).

*Alnarp Healing Gardens* as a health-oriented environment will in Yaloms philosophy support people’s responsibility and willing, expressed as existential theme of *freedom* (1980). Freedom will be found in the “wild aspects” of the garden, the old paths, moss-grown rocks and the wide spaces, where individuals can watch without participating. People may experience both responsibility and volition in the “common”, a green open space admitting of vistas and stay. This might support the healing process.

The *Fitness Centre* is a place of commitment to health behaviour. Participants – both patients and other users - express their wish for a superior and rigid structure (for example feedback from the machines or running tracks) that will help to devolve responsibility. Sometimes the seeking after routines appears to be ‘‘regressive’’, a behavior whereby people go back to an earlier stadium of development (Roessler, 2011). Once routines of exercising are established, participants can ‘‘grow’’ to take up a more self-determined ‘‘adult’’ position. Especially in the fitness center we have to be focused on the environment, which can support or hinder the participant. Emotional experiences require protected circumstances, empathy and awareness from the environment. Feeling protected, as a feeling of being safe in the training environment, supports movement, while a feeling of marginalization or threat will be contra productive. So the social and architectonical world of the training environment may work as a barrier or as a motivation. If participants experience the space as an attractive surrounding they can become ‘‘siblings’’ with a common attitude towards exercise, sharing the feeling of all being in the same boat.

It can be productive to apply a health oriented existential approach to these environmental cases. The analysis of the three cases was carried out from the subjective experiences of the author, an author who visited the three places under very different circumstances, for example as a visiting health psychologist (Alnarp), as a tourist (Potsdamer Platz), and as a female researcher in physical activity (fitness centre). These different contexts and cultures were not discussed as confounders, but they surely may have influenced the observations. However, the focus of this paper was not to deliver a complete theoretical approach, but to develop considerations and reflections out of participatory research, which might be applied to future environmental studies.

The observer is confronted with psychological contradictions that affect our relationship with space. Humans need to maintain and realise themselves, and thus distinguish themselves from others (isolation), but they have also the responsibility to answer. The fear of *isolation* affects your view of a space, as people will fear being both alone *and* with others. You are continually aware of the sense of *isolation* and *separation* engendered by our surroundings. Another psychological discrepancy is that we strive for *volition* and *responsibility*, but also for *change* and *challenge*.

Applying existential theory to three different cases helps to develop psychological relationships. A lot of other questions or approaches could have been relevant in the meeting between psychology, health and architecture. For example a discussion of what makes architecture attractive or repellant or what makes inhabitants feel a sense of attachment or identity could be stressed (Sallis et al., 1998).

It can be though concluded that three environments had different psychological qualities for an author, who met these environments under different circumstances (e.g. as a researcher, tourist and participator). For the theoretical development of further research we have to call attention of the *qualities* of a given space, of the *emotions* it triggers. Yalom’s approach helps to develop themes and to classify if an environment can encourage freedom or isolation or serve some other, more regulated purpose. A landscape and especially a cityscape can support the alienation (in Yalom’s language *isolation*) of human beings – here Alexander Mitscherlich (1971, 1972) did pioneering work, which during recent years has been followed up by the architect Anthony Vidler and others. If we want a space that does not promote standardised forms of movement and which is inviting and open, then there is a need for more knowledge on psychology. Furthermore, lack of opportunities for privacy and intimacy will compound the obstacles for women using the space. These themes have to be qualified in further research.

## 5. Limitations

Public Health usually uses quantitative research, for example in epidemiology. The limitation of a qualitative study is often a restricted insight to the experience of few individuals or groups. In addition, few individuals might have different emotions, when looking at the same architecture, depending on their individual background, experiences and interpersonal differences.

However, in this study, a qualitative approach was mainly chosen to start a discussion and to elaborate and exemplify subjective experiences, which may serve as basic knowledge for a broader and more quantitative study.

## 6. Conclusion

The combination of psychology, health and environment asks for more systematic qualitative and quantitative research. An existential and health oriented approach can support the establishment of phenomenological types, which can help to understand the relationship between environment and behaviour. It would be an illusion that Yaloms existential themes of anxiety can be found isolated in reality, but we could see them as a methodological way of sorting out the complexity of life. Many questions still remain to be answered – and to be posed.

## Footnotes

1 [Unsere leibliche Organisation ist die Form, unter der wir alles Körperliche auffassen. Ich werde nun zeigen, dass die Grundelemente der Architektur: Stoff und Form, Schwere und Kraft sich bestimmen nach den Erfahrungen, die wir an uns gemacht haben] (Wölfflin 1999/org.1886, p. 15).
